# Non-adherence to Haemodialysis, Interdialytic weight gain and cardiovascular mortality: a cohort study

**DOI:** 10.1186/s12882-019-1573-x

**Published:** 2019-11-06

**Authors:** Lianna G. G. Dantas, Mário de Seixas Rocha, José Andrade Moura Junior, Edson Luiz Paschoalin, Sandra R. K. P. Paschoalin, Constança M. Sampaio Cruz

**Affiliations:** 1 Clínica Senhor do Bonfim. Rua Plínio de Lima, 1. Monte Serrat, Salvador, BA Brazil; 2 Postgraduate Course in Medicine and Human Health, Bahia School of Medicine and Public Health, Salvador, Bahia Brazil; 3 Department of Multidisciplinary Research Hospital Santo Antonio, Social Works of Irmã Dulce, Salvador, Brazil

**Keywords:** Cardiovascular mortality, Haemodialysis, Interdialytic weight gain, Non-adherence, Survival

## Abstract

**Background:**

Patients with chronic kidney diseases (CKD) on haemodialysis (HD) have high morbidity and mortality rates, which are also due to the inherent risks associated with nephropathy. Non-adherence (NA) to the different demands of the treatment can have consequences for the outcome of patients undergoing HD; nevertheless, there are still doubts about such repercussions. This study was conducted to evaluate the association between NA to conventional HD and all-cause mortality and cardiovascular mortality.

**Methods:**

We prospectively evaluated mortality in a 6-year period in a cohort of 255 patients on HD in northeast Brazil. The evaluated parameters of NA to HD were interdialytic weight gain (IDWG) ≥ 4% of dry weight (DW), hyperphosphatemia and regular attendance at treatment, assessed as the correlation between the periods on HD completed and those prescribed. We used the Cox multivariate regression model to analyse survival and the predictors of all-cause mortality and cardiovascular mortality.

**Results:**

With a median follow-up period of 1493 days and a mortality rate of 9.1 per 100 people-years, there were 87 deaths, of which 54% were cardiovascular deaths. IDWG ≥4% of DW was associated with a risk of all-cause mortality however presenting a borderline outcome for cardiovascular mortality, with hazard ratios of 2.02 (CI 95% 1.17–3.49, *p* = 0.012) and 2.09 (CI 95% 1.01–4.35, *p* = 0.047), respectively. No significant association was found between other parameters of NA and mortality. Subgroup analysis showed that for patients with IDWG ≥4% of DW, malnutrition, age and diagnosis of cardiovascular and cerebrovascular diseases were associated with higher all-cause mortality.

**Conclusions:**

IDWG ≥4% of DW was identified as an independent predictor of all-cause mortality and demonstrated a borderline outcome for cardiovascular mortality in patients on conventional HD. The occurrence of excessive IDWG in the presence of malnutrition represented a significant increase in the risk of death, indicating a subgroup of patients with a worse prognosis.

## Background

The diagnosis of chronic kidney disease (CKD) with an indication for haemodialysis (HD) places several demands on patients to make important changes in their living habits including daily consumption of medications, limitations on the ingestion of food and water, and dependence on a machine for survival, generally with inflexible hours available for attending the dialysis sessions. Such demands may be perceived by the patients as an intrusion into their lives and may result in the patients not following the recommendations and prescriptions for the treatment, in other words, non-adherence (NA) [1].

The best criteria for determining NA to HD is still being debated as a result of research results assessing different demands of renal replacement therapy (RRT) [2–5]. Regular attendance at sessions, hyperphosphatemia and interdialytic weight gain (IDWG) have been investigated as indicators of NA to HD; nevertheless, there is no consensus and no undisputed findings that indicate NA to the RRT recommendations [2, 4, 6]. The prevalence of NA varies greatly in accordance with the region studied [7–9]. In Brazil, a high rate of reductions in HD sessions was identified (49%), and the prevalence of absences from HD sessions was similar to that reported in the USA (8%) [7, 9].

IDWG results from the consumption of salt and liquids between two HD sessions and reflects whether or not dietary and hydric restrictions particular to CKD dialysis have been followed [2, 7, 10, 11]. Patients undergoing HD receive nutritional guidance based on the RRT recommendations and the patient’s nutritional status to avoid hypervolemia. Excessive IDWG implies that the patient is being maintained in a hypervolemic state and is therefore susceptible to cardiovascular complications and haemodynamic instability due to rapid ultrafiltration (UF) during HD [12–15].

Despite all the advances in RRT, patients undergoing dialysis experience a survival rate inferior to the that of the general population in the same age group, and the role that NA plays in influencing the outcome of HD has been suggested as an additional risk factor for morbidity and mortality [2, 6, 16]. This study was designed to evaluate the association between NA to conventional HD and mortality. We tested the hypothesis that NA to the period of time prescribed, the frequency of the sessions (attendance criteria) and the dietary guidance (investigated as IDWG and hyperphosphatemia) are associated with an increased risk of all-cause mortality and cardiovascular mortality in patients undergoing HD.

## Methods

### Study design and subjects

A prospective cohort study was conducted that included patients on HD in a non-hospital service in Salvador, the northeastern region of Brazil. The sample consisted of adults without cognitive or psychiatric impairment who had been on HD for at least 3 months, to avoid inclusion of patients with acute renal injury and patients without knowledge of the nutritional guidelines, and still in initial clinical stabilization in the RRT. Of the 269 patients undergoing haemodialysis at the clinic, 8 were excluded due to the impossibility of obtaining consent because of physical limitations and the absence of authorized representatives, and 6 refused to participate in the study. Between November 2011 and January 2012, 255 patients joined the cohort and were followed until November 2017, at which point they were continuing with the HD in the service, the outcome had transpired or they were lost to follow-up. All had been given prescriptions for three sessions of haemodialysis per week with a duration of 4 h per session, using dialyzers with biocompatible membranes and variable dialysate sodium of 136 to 142 mEq/L. The initial data collection was part of the master’s project of the author started in 2011 and the baseline data were collected in the same month for all patients [9, 17]. The study was in compliance with the Helsinki declaration, and the protocol was approved by the Research Ethics Committee of the Spanish Hospital and Bahia School of Medicine and Public Health.

### Data collection

Sociodemographic data of interest (age, race, sex, civil status, schooling level), RRT data (period of dialysis, vascular access, residual renal function, history of parathyroidectomy and kidney transplant), clinical data, aetiology of CKD and diagnosis of diabetes mellitus (DM), systemic arterial hypertension (SAH) and cardiovascular disease or cerebrovascular were collected at the beginning of the study. The diagnoses of coronary arterial disease (history of acute myocardial infarction, myocardial revascularization or coronary angioplasty), congestive cardiac failure with low ejection fraction, cerebrovascular disease (history of transient ischaemic stroke or cerebrovascular ischaemic or haemorrhagic accident—CVA) and peripheral arterial occlusive disease (history of non-traumatic amputation of extremities or symptomatic ischaemic disease confirmed by Doppler or arteriography) were grouped and considered to represent clinical evidence of cardiovascular or cerebrovascular diseases for analysis in the study.

### Exposure variables, outcome and covariates

The primary outcome of interest was all-cause mortality being cardiovascular mortality considered as a secondary outcome. IDWG, hyperphosphatemia and regular attendance at treatment were assessed as parameters of NA to HD. IDWG was calculated as the difference between the pre-HD weight and the weight registered after the previous session; the average of the sessions in a month were registered and assessed as absolute IDWG and relative IDWG (absolute IDWG divided by dry weight [DW]). IDWG as an absolute value was analysed as a continuous variable. Relative IDWG was classified into three groups: < 3%, 3 to 3.99% and ≥ 4% of DW. IDWG > 4% of DW was the range considered to be indicative of excessive IDWG and corresponded to the threshold value used in a prior study of Mi Jung Lee [15]. DW was determined and adjusted by the assistant nephrologist in the context of the clinical condition, intradialytic and post-HD symptoms. The average phosphorus (P) level for 3 consecutive months was calculated and considered as hyperphosphatemia when greater than 5.5 mg/dL. For the analysis, reductions in regular attendance at treatment and absences from dialysis sessions over 3 consecutive months from the beginning of the study were computed, and the relationship between the period of HD completed and the period prescribed was also computed. NA was considered when the patient did not perform 100% of the prescribed HD period, independent of the reasons for absence from or reductions in the sessions.

The UF rate was considered to be the volume removed during the HD session divided by the DW and the duration of the dialysis; 12 consecutive sessions were assessed, and the average rate of UF was calculated. All of the samples were collected before the first HD session of the week for the laboratory dosages of interest and after dialysis to calculate the fractional clearance of the urea (spKt/V) using the Daurgirdas 2nd generation formula [18]. Missing laboratory results were repeated and we had no lack of information from the NA variables to haemodialysis. The urinary volume for the 24 h was quantified during the longest interdialytic period for evaluation of the residual renal function (RRF) and classified as anuria when diuresis was < 100 ml per day.

Patient deaths during follow-up were recorded according to death certificates or hospital records. These included deaths that occurred on or outside haemodialysis. If the reason for death was not clarified, it was recorded as death of undetermined cause. Cardiovascular mortality was defined as sudden cardiac death, death from acute myocardial infarction, heart failure, acute lung edema, arrhythmias, stroke or other fatal ischemic events.

### Statistical analysis

To calculate the sample size for our study we considered a 24% reduction in the relative risk of overall mortality related to improvement in the proportion of adherence time for haemodialysis, which was identified in the Kimmel study, with 5% accuracy and 80% power [8]. Sociodemographic, clinical and laboratory characteristics at the beginning of the study were described as absolute and relative frequencies (percentages) when qualitative and as the mean ± standard deviation (SD) when continuous variables with a normal distribution or median and interquartile range (IQR) for a non-normal distribution. For these analyses, we had no missing covariate values. The prevalence of NA was expressed in percentages with the respective confidence intervals of 95% (CI 95%). The groups were compared using the chi-square test, t-test for independent samples or analysis of variance (ANOVA) with the post hoc Bonferroni test.

The survival time of each patient was considered as the interval between the beginning of the study and death or the end of the observation period. The survival univariate curve was researched using the Kaplan-Meier analysis with the log-rank test and the Cox proportional hazard model to evaluate mortality predictors. The variables with biological plausibility and *p* <  0.20 in the univariate analysis were included in the multivariate logistic regression model, and the results were expressed as the hazard ratio (HR) with the respective 95% CI. The existence of colinearity between the variables that were included in the multivariate regression model was investigated. The level of significance was set at two-tailed *p* value of < 0.05. All analyses were performed using SPSS version 20.0 (SPSS Inc., Chicago, Ill., USA).

## Results

### Cohort characteristics and non-adherence measures

The sociodemographic and clinical characteristics of the patients at the beginning of the cohort are provided in Table 1. The mean age was 50 ± 13.1 years, and the proportion of men was higher (62.7%) than that of women; other characteristics included non-white (85.5%), married (62%) and on haemodialysis with arteriovenous fistula (81.7%). The median period of haemodialysis treatment was 39 months (17–76 months). SAH was the most frequent aetiology of nephropathy (28.6% of the cases), followed by glomerulonephritis (16.5%) and DM (14.5%). A total of 39.2% of the patients were smokers, and of these, 91% abandoned the habit after beginning the RRT. A total of 77.3% of the patients underwent their treatment through the public health service (PHS). Table 2 demonstrates the baseline characteristics of the patients in accordance with the assessed NA parameters. The patients with IDWG ≥4% of DW underwent HD for longer periods, were younger and had higher Kt/V than patients with IDWG < 3% of DW, as well as a higher prevalence of anuria and SAH, and the highest average of phosphate than the other groups. Of the patients with excessive IDWG, 5.2% had a catheter with vascular access for HD versus 14.7% of patients with IDWG < 3% of DW; nevertheless, this difference was not significant (*p* = 0.05). Sex, race, body mass index (BMI), diagnosis of cardiovascular or cerebrovascular disease and DM, haemoglobin and albumin results did not differ among the three groups. The average UF rate was significantly different among the three investigated relative IDWG ranges, with an average of 12.5 ± 2.95 ml/h/kg in the group with excessive IDWG versus 6.18 ± 2.60 in the group with IDWG < 3% of DW. Those patients with phosphorus levels > 5.5 mg/dL were undergoing dialysis for a longer period, had lower average spKt/V, higher average BMI, higher frequency of anuria and of parathyroid hormone (PTH) ≥ 600 pg/mL and a higher UF average in HD than patients with phosphorus levels ≤5.5 mg/dL. Patients undergoing dialysis for less time than the amount prescribed per month were predominantly non-diabetic, non-white and had a higher frequency of PTH ≥ 600 pg/mL.

**Table 1:** Sociodemographic and clinical characteristics of the sample

	*n* = 255 (%)
Age, years	50 ± 13.1
Age > 65 years	30 (11.8)
Race (self-referred)	
White	37 (14.5)
Non-white	218 (85.5)
Sex	
Male	160 (62.7)
Civil status	
Married	158 (62)
Residents in Salvador	217 (85.1)
Aetiology of CKD	
Indeterminate	65 (25.5)
Systemic Arterial Hypertension	73 (28.6)
Glomerulonephritis	42 (16.5)
Diabetes mellitus	37 (14.5)
Polycystic Kidney Disease	13 (5.1)
Vasculitis	6 (2.4)
Other pathologies	19 (7.5)
Time on haemodialysis, months	39 (17–76)
Haemodialysis by catheter	21 (8.3)
Anuric	127 (49.8)
Urine volume, mL/day	150 (0–500)
Body mass index, Kg/m^2^	23.8 ± 4.3
Smoking (presently or prior)	100 (39.2)
Background of parathyroidectomy	20 (7.8)
Pre-dialysis follow-up	96 (37.6)
Living alone	16 (6.3)
Schooling level up to elementary education	118 (46.3)
Family income per capita, USD	181 (109–272.5)
< 2 USD per person/day	32 (12.5)
Treatment through the public health service national	197 (77.3)
Comorbid disease	
Diabetes Mellitus	41 (16.1)
Cardiovascular or cerebrovascular disease	84 (32.9)
Systemic Arterial Hypertension	231 (90.6)

Qualitative variables expressed in absolute values (%) and quantitative values as mean ± SD or median (1st-3rd quartile ranges) for continuous variables in accordance with distribution

*USD* U.S. dollars

**Table 2:** Basal demographic and clinical characteristics of patients in accordance with non-adherence to haemodialysis parameters

		Interdialytic weight gain				% Period of HD			Phosphorus (mg/dL)		
	Global	< 3%DW	3–3.99%DW	≥ 4%DW		100%	< 100%		≤ 5.5	> 5.5	
	255 (100)	75 (29.5)	83 (32.5)	97 (38)	p	123 (48.2)	132 (51.8)	p	149 (58.4)	106 (41.6)	p
Age, years	50 ± 13.1	54.4 ± 13.8	50 ± 12.6	46.4 ± 12	< 0.001	51.7 ± 13.2	48.3 ± 12.8	0.29	50.6 ± 13.7	49 ± 12.3	0.35
Sex (female)	95 (37.2)	23 (30.7)	33 (39.8)	39 (40.2)	0.37	47 (38.2)	48 (36.4)	0.76	57 (38.3)	38 (35.8)	0.69
Race (non-white)	218 (85.5)	63 (84)	68 (81.9)	87 (89.7)	0.3	99 (80.5)	119 (90.2)	0.029	130 (87.2)	88 (83)	0.34
Diabetes Mellitus (yes)	41 (16.1)	11 (14.7)	16 (19.3)	14 (14.4)	0.62	26 (21.1)	15 (11.4)	0.034	22 (14.8)	19 (17.9)	0.49
Systemic arterial hypertension (yes)	231 (90.6)	62 (82.7)	79 (95.2)	90 (92.8)	0.017	115 (93.5)	116 (87.9)	0.12	136 (91.3)	95 (89.6)	0.65
Cardiovascular or cerebrovascular disease (yes)	84 (32.9)	27 (36)	28 (33.7)	29 (29.9)	0.68	46 (37.4)	38 (28.8)	0.14	51 (34.2)	33 (31.1)	0.6
Smoking (yes)	100 (39.2)	32 (42.7)	30 (36.1)	38 (39.2)	0.70	47 (38.2)	53 (40.2)	0.75	54 (36.2)	46 (43.4)	0.24
Time on HD (months)											
< 24	91 (35.7)	30 (40)	34 (41)	27 (27.8)	0.045	48 (39)	43 (32.6)	0.53	63 (42.3)	28 (26.4)	0.03
24–59	81 (31.8)	29 (38.7)	22 (26.5)	30 (30.9)		36 (29.3)	45 (34.1)		44 (29.5)	37 (34.9)	
≥ 60	83 (32.5)	16 (21.3)	27 (32.5)	40 (41.2)		39 (31.7)	44 (33.3)		42 (28.2)	41 (38.7)	
Vascular access (catheter)	21 (8.3)	11 (14.7)	5 (6)	5 (5.2)	0.053	12 (9.8)	9 (6.8)	0.39	13 (8.7)	8 (7.5)	0.73
Status for kidney transplant (apt)	37 (14.5)	9 (12)	13 (15.7)	15 (15.5)	0.76	17 (13.8)	20 (15.2)	0.76	21 (14.1)	19 (17.9)	0.40
Schooling level up to elementary education	118 (46.3)	36 (48)	41 (49.4)	41 (42.3)	0.59	57 (46.3)	61 (46.2)	0.98	72 (48.3)	46 (43.4)	0.43
BMI (Kg/m^2^)	23.8 ± 4.3	24.4 ± 4.64	24.1 ± 4.16	23.1 ± 4.22	0.095	24.2 ± 4.25	23.5 ± 4.43	0.2	23.2 ± 4.3	24.7 ± 4.3	0.009
Anuria (yes)	127 (49.8)	20 (26.7)	42 (50.6)	65 (67)	< 0.001	61 (49.6)	66 (50)	0.94	65 (43.6)	62 (58.5)	0.01
spKt/V	1.36 ± 0.34	1.28 ± 0.31	1.37 ± 0.34	1.41 ± 0.34	0.03	1.39 ± 0.33	1.33 ± 0.34	0.19	1.40 ± 0.34	1.30 ± 0.33	0.027
Ultrafiltration (ml/h/Kg)	9.49 ± 3.73	6.18 ± 2.60	8.93 ± 2.41	12.5 ± 2.95	< 0.001	9.42 ± 3.44	9.55 ± 4.0	0.78	9.0 ± 3.86	10.0 ± 3.48	0.03
Serum phosphorus (mg/dL)	5.44 ± 1.67	4.94 ± 1.52	5.16 ± 1.49	6.06 ± 1.74	< 0.001	5.44 ± 1.64	5.43 ± 1.70	0.96			
Parathyroid hormone ≥600 pg/mL	55 (21.6)	15 (20)	14 (16.9)	26 (26.8)	0.25	19 (15.4)	36 (27.3)	0.022	25 (16.8)	30 (28.3)	0.027
Serum albumin (g/dL)	3.58 ± 0.46	3.58 ± 0.52	3.55 ± 0.42	3.61 ± 0.45	0.71	3.54 ± 0.45	3.62 ± 0.48	0.19	3.58 ± 0.47	3.59 ± 0.45	0.86
Haemoglobin (g/dL)	11.1 ± 1.45	10.7 ± 1.41	11.2 ± 1.41	11.2 ± 1.49	0.065	11 ± 1.57	11.1 ± 1.34	0.59	10.9 ± 1.46	11.3 ± 1.42	0.07
Period of HD performed /prescribed < 100%		42 (56)	38 (45.8)	52 (53.6)	0.39				75 (50.3)	57 (53.8)	0.58

Data expressed as mean ± SD for continuous variables and n (%) for categorical variables

*DW* dry weight, *IDWG* interdialytic weight gain, *HD* haemodialysis, *BMI* body mass index, *spKt/V* Fractional clearance of urea

### Mortality and non-adherence to haemodialysis

There were 87 deaths during 347,636 person-years of follow-up, with a mortality rate of 9.1 per 100 people-years. The average follow-up duration was 1493 days (535–2191), and 54% of the deaths occurred due to cardiovascular causes, of which 14.9% were sudden deaths, 13.8% were acute myocardial infarctions, and 13.8% were due to stroke; the remaining deaths were due to acute oedema of the lung, arrhythmias or mesenteric ischaemia.

Figure 1 demonstrates the survival curve for cardiovascular mortality by relative IDWG range. Cardiovascular mortality was significantly higher in the group of patients with IDWG ≥4% of DW (*p* = 0.041) than in patients with IDWG < 3% of DW. The same analysis was performed to assess all-cause mortality, but no significant difference (*p* = 0.15) was observed in the univariate analysis (the Kaplan-Meier graph is attached as Additional file 1: Figure S1).

Table 3 demonstrates the Cox regression analysis to identify the independent predictors of all-cause mortality; IDWG ≥4% of DW was associated with an increase in the risk of mortality of 102% compared with IDWG under 3% of DW. Age, clinically evident cardiovascular or cerebrovascular diseases and malnutrition were independent predictors for the outcome. The remaining parameters of NA assessed were not identified as independent predictors of all-cause mortality.

**Table 3:** Cox proportional hazard model for all-cause mortality

Risk factors	Unadjusted		Multivariate	
	HR (CI 95%)	*p*	HR (CI 95%)	*p*
Age (years)	1.04 (1.03–1.06)	< 0.001	1.04 (1.02–1.06)	< 0.001
Time in haemodialysis (years)	1.04 (0.99–1.09)	0.11	1.04 (0.97–1.11)	0.21
Cardiovascular or cerebrovascular disease (yes)	2.28 (1.50–3.47)	< 0.001	1.80 (1.16–2.79)	0.009
Diabetes Mellitus (yes)	1.83 (1.13–2.97)	0.014	0.66 (0.39–1.11)	0.12
Smoking (presently or prior)	1.46 (0.96–2.23)	0.075	1.04 (0.66–1.62)	0.85
Anuria (yes)	1.39 (0.91–2.12)	0.126	1.57 (0.96–2.55)	0.07
Vascular access (catheter)	1.71 (0.86–3.42)	0.12	0.62 (0.29–1.28)	0.20
Status for kidney transplant (not apt)	1.73 (1.05–2.86)	0.03	1.70 (0.59–4.89)	0.32
BMI between 18.5–24.9 Kg/m^2^	1 (reference)	0.015	1 (reference)	
BMI between 25 and 29.9 Kg/m^2^	1.54 (0.94–2.53)	0.085	1.45 (0.88–2.38)	0.14
BMI ≥ 30 Kg/m^2^	1.14 (0.51–2.55)	0.73	0.94 (0.41–2.11)	0.87
BMI < 18.5 Kg/m^2^	2.83 (1.54–5.22)	0.001	2.24 (1.19–4.20)	0.012
Serum albumin (g/dL)	0.65 (0.41–1.03)	0.07	0.81 (0.50–1.31)	0.39
% period of HD performed/prescribed	1.05 (0.99–1.13)	0.09	1.00 (0.95–1.06)	0.77
IDWG < 3% of DW	1 (reference)	0.17	1 (reference)	
IDWG 3–3.99% of DW	0.81 (0.46–1.44)	0.29	0.89 (0.49–1.61)	0.69
IDWG ≥4% of DW	1.31 (0.79–2.17)	0.18	2.02 (1.17–3.49)	0.012

*HR* Hazard ratio, *CI* confidence interval, *BMI* body mass index, *IDWG* interdialytic weight gain, *DW* dry weigh

Other variable assessed in the univariate analysis: sex, race, systemic arterial hypertension, background of parathyroidectomy, spKt/V, Parathormone ≥600 pg/mL, Serum phosphorus > 5.5 mg/dL, Ultrafiltration (ml/h/Kg)

IDWG ≥4% of DW presented borderline result in multivariate analysis for cardiovascular mortality with hazard ratios of 2.09 (CI 95% 1.01–4.35, *p* = 0.047). Clinical predictors for cardiovascular mortality were malnutrition, dialysis using a catheter, DM and a clinically evident background of cardiovascular or cerebrovascular diseases (Table 4). IDWG analysed as an absolute value was not associated with mortality, and there was no colinearity between the variables that were included in the multiple regression model for all-cause mortality and cardiovascular mortality.

**Table 4:** Cox proportional model hazard for cardiovascular mortality

Risk factors	Unadjusted		Multivariate	
	HR (CI 95%)	*p*	HR (CI 95%)	*p*
Age (years)	1.03 (1.01–1.06)	0.005	1.01 (0.98–1.04)	0.26
Cardiovascular or cerebrovascular disease (yes)	3.48 (1.94–6.24)	< 0.001	3.57 (1.94–6.55)	< 0.001
Diabetes Mellitus (yes)	2.80 (1.53–5.13)	0.001	3.45 (1.80–6.61)	< 0.001
Anuria (yes)	1.53 (0.86–2.74)	0.14	1.52 (0.77–3.00)	0.21
Vascular access (catheter)	2.19 (0.93–5.17)	0.07	2.63 (1.04–6.64)	0.04
Status for kidney transplant (not apt)	3.23 (0.78–13.3)	0.10	1.86 (0.42–8.12)	0.40
Ultrafiltration (ml/h/Kg)	1.05 (0.94–1.17)	0.17	1.05 (0.94–1.17)	0.37
BMI between 18,5–24.9 Kg/m^2^	1 (reference)	0.16	1 (reference)	0.02
BMI between 25 and 29.9 Kg/m^2^	1.44 (0.74–2.80)	0.27	1.78 (0.91–3.51)	0.09
BMI ≥ 30 Kg/m^2^	0.84 (0.25–2.81)	0.78	0.80 (0.23–2.77)	0.73
BMI < 18.5 Kg/m^2^	2.47 (1.06–5.77)	0.03	3.67 (1.48–9.06)	0.005
% period of HD performed/prescribed (< 100%)	0.63 (0.35–1.13)	0.12	0.86 (0.46–1.63)	0.66
IDWG < 3% of DW	1 (reference)	0.06	1 (reference)	0.005
IDWG 3–3.99% of DW	0.70 (0.30–1.63)	0.42	0.65 (0.27–1.52)	0.32
IDWG ≥4% of DW	1.63 (0.82–3.25)	0.16	2.09 (1.01–4.35)	0.047

*HR* Hazard ratio, *CI* confidence interval, *BMI* body mass index, *IDWG* interdialytic weight gain, *DW* dry weight

Other variables assessed in the univariate analysis: sex, race, time on hemodialysis, smoking, systemic arterial hypertension, background of parathyroidectomy, Serum albumin, spKt/V, Parathormone ≥600 pg/mL, Serum phosphorus > 5.5 mg/dL

### Subgroup analysis

Table 5 demonstrates the Cox regression analysis for all-cause mortality in the subgroup of 97 patients with IDWG ≥4% of DW. It was found that malnutrition represented a significant increase in the risk of death (306%) in relation to the eutrophic state. Age and diagnosis of cardiovascular and cerebrovascular diseases were also independent predictors of mortality, with HR values of 1.05 and 2.74, respectively.

**Table 5:** Cox proportional model hazard for all-cause mortality in the subgroup with IDWG ≥4% of DW

Risk factors	Unadjusted		Multivariate	
	HR (CI 95%)	*p*	HR (CI 95%)	*p*
Age (years)	1.06 (1.02–1.09)	< 0.001	1.05 (1.01–1.08)	0.002
Cardiovascular or cerebrovascular disease (yes)	3.39 (1.81–6.33)	< 0.001	2.74 (1.42–5.27)	0.003
Diabetes Mellitus (yes)	1.74 (0.80–3.79)	0.15	1.09 (0.43–2.74)	0.84
Vascular access (catheter)	3.13 (0.96–10.23)	0.05	2.35 (0.63–8.22)	0.18
BMI between 18,5–24.9 Kg/m^2^	1 (reference)	0.045	1 (reference)	
BMI between 25 and 29.9 Kg/m^2^	1.18 (0.52–2.67)	0.68	0.85 (0.37–1.95)	0.70
BMI ≥ 30 Kg/m^2^	0.93 (0.21–3.98)	0.92	1.26 (0.28–5.53)	0.96
BMI < 18.5 Kg/m^2^	3.02 (1.38–6.63)	0.006	3.06 (1.39–6.76)	0.005

*HR* Hazard ratio, *CI* confidence interval, *BMI* body mass index, *IDWG* interdialytic weight gain, *DW* dry weight

Other variables assessed in the univariate analysis: sex, race, time on hemodialysis, smoking, systemic arterial hypertension, background of parathyroidectomy, Serum albumin, spKt/V, Parathormone ≥600 pg/mL, Serum phosphorus > 5.5 mg/dL, Ultrafiltration (ml/h/Kg)

## Discussion

This study broadly assessed the association between certain NA to HD parameters and mortality and found that IDWG ≥4% of DW was an independent predictor of all-cause mortality in HD patients. The other investigated NA parameters were not predictors of mortality in this cohort. Various studies have assessed IDWG as an indicator of adherence to RRT and its associations with cardiovascular complications and mortality in HD and the nutritional state of the patient, though variable relative and absolute IDWG gradients were applied, and conflicting results were reported [2, 3, 6, 10, 11, 15, 19–21].

In this cohort, patients with excessive IDWG were younger and had a higher prevalence of SAH than the group with IDWG < 3% of DW. SAH may be the consequence of hydric overload related to excessive IDWG during a long period of RRT; nevertheless, it was not possible to confirm this hypothesis because it was beyond the scope of the present work. Five decades ago, Thomson presented the concept that pressure control could be obtained with a reduction in extracellular volume, and since then, various studies have demonstrated the association between SAH and IDWG and effective pressure control with gradual reduction of DW in the HD sessions [22, 23]. Anuria was more prevalent in the group with excessive IDWG compared with the group with IDWG < 3% of the DW (67% versus 26.7%) and may have resulted from repeated episodes of intradialytic hypotension in an attempt to achieve euvolemia. Prior studies have demonstrated such an association, as well as an inverse correlation between the duration of the RRT and the reduction of the RRF [24–26]. RRF helps in the maintenance of the hydric balance, pressure control, and clearance of middle molecules and protein-bound solutes and therefore contributes to metabolic homeostasis. In this manner, the preservation of RRF has been the object of attention in recent years [27]. The faster loss of RRF is one of the mechanisms through which excessive IDWG could have an adverse effect on the progress of the patient.

Patients with excessive IDWG had a higher average of Kt/V and phosphorus; nevertheless, the average albumin and BMI were similar among the 3 investigated ranges of IDWG. In a cohort in Taiwan, the effect of IDWG on BMI was studied, and results of albumin, creatinine, urea and phosphorus were obtained; in the 255 patients evaluated, no differences were found in the nutritional markers between the categories of relative IDWG or variations in the relative IDWG values and BMI throughout the 12-month follow-up [10]. Other authors have shown that IDWG is constant for each patient and is influenced by nutritional habits, environmental factors, self-care and patient response to the demands of HD [5, 6, 11].

In initial studies established to assess the consequences of NA to HD, the acceptable limits of IDWG and P were more permissive [2]. In 1998, Leggat et al. used IDWG > 5.7% of DW as a criterion to indicate excessive gain, and this was associated with an increase in the relative risk of mortality [2]. In the Dialysis Outcomes and Practice Patterns Study, IDWG ≥4% and ≥ 5.7% of DW were associated with a higher risk of hospitalization due to hypervolemia and a higher mortality, respectively, and furthermore, evidenced a decline in the relative IDWG throughout phases two to five of the study [6]. Various publications also found associations between lower relative IDWG ranges and mortality [3, 15, 21, 28]. In the cohort of Cabrera et al., with 39,256 patients on haemodialysis, IDWG > 3.5% of DW was associated with the following outcomes: myocardial infarction (HR 1.18), cardiovascular mortality (HR 1.23), death for all causes (HR 1.26) in addition to hospitalizations for cardiac complications [20].

Our findings regarding the increase of all-cause mortality (HR 2.02) and a borderline outcome for cardiovascular mortality in patients on HD with IDWG ≥4% of DW versus IDWG < 3% of DW may be partially explained. Excessive IDWG causes periods of hypervolemia that foments adaptive mechanisms, increasing pressure levels, left ventricular hypertrophy, arterial rigidity and adverse cardiovascular events [14]. Higher IDWG results in HD with a higher rate of ultrafiltration and intravascular contraction, which could precipitate intradialytic hypotension and a reduction in tissue perfusion leading to ischaemic complications and transitory abnormalities in myocardial contractility during and post-HD session, apart from the premature termination of dialysis and the mistaken perception that DW was obtained [29–31]. Although not significant, *p* value was borderline and possibly with a larger sample size could demonstrate an association between IDWG, mortality and cardiovascular complications. Mi Jung Lee et al. evaluated IDWG and cardiovascular outcomes in 1013 patients undergoing HD in South Korea and found that IDWG ≥4% of DW was an independent predictor of mortality or hospitalization due to cardiovascular or cerebrovascular events (HR 1.93; *p* = 0.04) [15].

A high rate of UF depends on a high IDWG, shorter duration of the session, lower body weight, or a combination of these variables [32]. In our study, patients with excessive IDWG had higher UF averages in the sessions, although the UF rate was not a predictor of mortality in the univariate and multivariate analyses. A high UF rate in HD was associated with higher mortality in the post hoc analysis of the HEMO study, and UF values above 13 ml/kg/hour were associated with a significant increase in the risk of all-cause mortality (59%) and cardiovascular mortality (71%) [30, 33].

In this study, age was an independent predictor of general mortality but was not associated with mortality due to cardiovascular causes. The role of cardiovascular diseases is known as the main cause of mortality in young people undergoing dialysis and has less relevance in elderly people, possibly due to the reduction in immune response related to ageing and a greater occurrence of infections in this population and, consequently, deaths [34–36]. DM was not a predictor of all-cause mortality in this study but was an independent predictor of cardiovascular mortality. The reason behind such findings are not clear, but our hypothesis is that the presence of subclinical vascular diseases in diabetic patients was a reason for higher mortality due to the cardiovascular events that we identified. Nevertheless, we do not have the results of complementary exams for tracing the subclinical cardiovascular lesions of these patients and the consequent confirmation of this hypothesis.

In our cohort, BMI < 18.5 kg/m^2^ was an independent predictor of all-cause and cardiovascular mortality. In the subgroup analysis, patients with IDWG ≥4% of DW had a significant increase in mortality in the presence of low weight, according to the category of BMI adopted by the World Health Organization [37]. BMI is a widely used nutritional parameter for risk assessment and nutritional scores of patients on HD [38, 39]. Unintentional reductions in BMI, represented by a decrease of more than 5% of DW in a period of 3–6 months, are predictive of a risk of protein-energy malnutrition in patients on HD [38]. In CKD, the lowest rate of survival on dialysis is found in patients with lower BMI values in the context of malnutrition and inflammation, possibly due to the action of inflammatory cytokines causing protein catabolism and anorexia [13, 39–42]. Thus, malnutrition is not a cause of morbidity and mortality but an indication of coexisting pathology that has repercussions on survival [43].

Some studies have shown that higher IDWG correlates with better nutritional status however, the relationship between these two variables remains controversial [11, 44, 45]. Yang et al., when evaluating the relationship between IDWG and nutritional markers, identified that the highest relative IDWG was associated with the worst nutritional status only in the elderly group [45]. In a multicentre international cohort that evaluated the evolution of clinical and laboratory indicators, patients had constant relative IDWG, but this declined significantly weeks before death in some populations [46]. We believe that IDWG ≥4% of DW in the context of malnutrition may represent excessive consumption of sodium and liquids, with a lower caloric and protein intake than the patient needs. This clinical scenario indicates a poor prognosis that demands a specific approach in clinical practice and possible improvement in the outcomes.

There are various limitations to this study. The history of cardiovascular or cerebrovascular disease considered was a criterion of evident clinical disease and was itself complicated and therefore not very sensitive; however, it does point out the need for greater care in this group of patients who are at very high risk, as well as the need for investigation of the presence of subclinical cardiovascular diseases in asymptomatic patients. The sample was predominantly non-white and therefore does not reflect the reality of many regions in Brazil or of many countries but represents the local reality of a very mixed race population. The mortality disclosed was lower than that reported in other publications in Brazil and in many countries, and such findings may be due to the reduced prevalence of DM in this cohort, as well as the exclusion of patients in the first three months of HD, a period known for higher mortality in dialysis [47, 48]. However, this cohort has the strength of an interval observation period of six years, with patients under HD for variable periods and, therefore, with diverse clinical situations due to the base pathologies and the complications of nephropathy. Our findings are clinically relevant and reproduce results from other HD populations that present a distinct ethnic group and human development index, thus increasing their external validity. Despite the importance of knowing the ideal ranges of IDWG associated with the lower morbidity and mortality of the haemodialysis patient as well as the role of the other parameters of NA, there aren’t publications in Brazil about NA in HD and clinical outcomes.

## Conclusions

In conclusion, we demonstrated that IDWG ≥4% of DW is an independent predictor of all-cause mortality in patients undergoing HD. The occurrence of excessive IDWG in the presence of malnutrition represented a significant increase in the risk of death, indicating a subgroup of patients with a worse prognosis. For patients undergoing HD, strategies to avoid excessive interdialytic weight gain without impairing the maintenance of adequate nutritional status, associated with a higher frequency of haemodialysis or longer duration of the session to control hypervolemia, may be proposed for better survival.

## Supplementary information

**Additional file 1: Figure S1.** Kaplan-Meier survival curves for all-cause mortality by relative interdialytic weight gain range. Kaplan-Meier survival analysis for all-cause mortality by relative interdialytic weight gain range. Survival estimates showed no significant difference in all-cause mortality (Log rank; *p* = 0.15).

## Data Availability

Data will be available upon request from the corresponding author (LGGD).

## Abbreviations

BMI: Body mass index
CI: Confidence intervals
CKD: Chronic kidney disease
CVA: Cerebrovascular accident
DM: Diabetes Mellitus
DW: Dry weight
HD: Hemodialysis
HR: Hazard ratio
IDWG: Interdialytic weight gain
IQR: Interquartile range
NA: Non-adherence
P: Phosphorus
PHS: Public health service
PTH: Parathyroid hormone
RRF: Residual renal function
RRT: Renal replacement therapy
SAH: Systemic arterial hypertension
SD: Standard deviation
spKt/V: Fractional clearance of the urea
UF: Ultrafiltration
USD: U.S. dollars
