# Supplementary material for: Non-adherence to Haemodialysis, Interdialytic weight gain and cardiovascular mortality: a cohort study
Source: BMC Nephrol. 2019 Nov 6;20:402. doi: 10.1186/s12882-019-1573-x (PMC6836324; doi:10.1186/s12882-019-1573-x)
Supplement: Supplementary file 1 — Additional file 1: Figure S1. Kaplan-Meier survival curves for all-cause mortality by relative interdialytic weight gain range. Kaplan-Meier survival analysis for all-cause mortality by relative interdialytic weight gain range. Survival estimates showed no significant difference in all-cause mortality (Log rank; p = 0.15). [file 12882_2019_1573_MOESM1_ESM.docx]

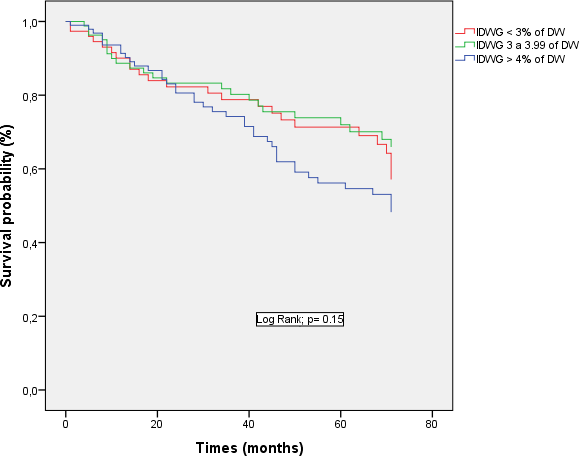


Figure S1

Kaplan-Meier survival estimates for all-cause mortality by relative interdialytic weight gain range
